# Sodium-Selective Channelrhodopsins

**DOI:** 10.3390/cells13221852

**Published:** 2024-11-08

**Authors:** Ariel Coli, Shiqiang Gao, Lars Kaestner

**Affiliations:** 1 Dynamics of Fluids, Experimental Physics, Saarland University, 66123 Saarbrücken, Germany; ariel.coli@uni-saarland.de; 2 Department of Neurophysiology, Physiological Institute, University of Würzburg, 97070 Würzburg, Germany; gao.shiqiang@uni-wuerzburg.de; 3 Theoretical Medicine and Biosciences, Medical Faculty, Saarland University, 66421 Homburg, Germany

**Keywords:** channelrhodopsins, optogenetics, sodium-selectivity, ChR2, channelrhodopsin variants, red blood cells

## Abstract

Channelrhodopsins (ChRs) are light-gated ion channels originally discovered in algae and are commonly used in neuroscience for controlling the electrical activity of neurons with high precision. Initially-discovered ChRs were non-selective cation channels, allowing the flow of multiple ions, such as Na^+^, K^+^, H^+^, and Ca^2+^, leading to membrane depolarization and triggering action potentials in neurons. As the field of optogenetics has evolved, ChRs with more specific ion selectivity were discovered or engineered, offering more precise optogenetic manipulation. This review highlights the natural occurrence and engineered variants of sodium-selective channelrhodopsins (NaChRs), emphasizing their importance in optogenetic applications. These tools offer enhanced specificity in Na^+^ ion conduction, reducing unwanted effects from other ions, and generating strong depolarizing currents. Some of the NaChRs showed nearly no desensitization upon light illumination. These characteristics make them particularly useful for experiments requiring robust depolarization or direct Na^+^ ion manipulation. The review further discusses the molecular structure of these channels, recent advances in their development, and potential applications, including a proposed drug delivery system using NaChR-expressing red blood cells that could be triggered to release therapeutic agents upon light activation. This review concludes with a forward-looking perspective on expanding the use of NaChRs in both basic research and clinical settings.

## 1. Introduction

Channelrhodopsins (ChRs) are light-gated ion channels originally discovered in eukaryotic algae where, through ion flux, they are responsible for the phototactic movement of the algae. Presently, they are principally used as tools in neuroscience to control the electrical activity of neurons through high spatiotemporal precision [1].

The ability of ChRs to induce rapid changes in membrane potential, though sodium (Na^+^) influx to a great extent, is vital for experiments requiring high temporal resolution [2]. The speed at which these channels open and close upon light activation allows researchers to manipulate neuronal activity on a millisecond timescale, which is necessary for dissecting the fast dynamics of neural processes and synaptic transmission [3]. In this respect, the manipulation of the membrane potential needs to be at least as fast as the fluorescence-based read-out of the membrane potential, which can be challenging for genetically encoded voltage sensors [4].

These ChRs have been intensely studied and modified to improve characteristics and express certain properties. In this review, we focus on one specific branch of channelrhodopsins, whose aim is to selectively channel Na^+^ across the cell membrane. These channels have been either discovered in nature with an elevated affinity to Na^+^ or engineered to enhance their Na^+^ permeability, thus, providing powerful tools for optogenetic applications [2,3].

Furthermore, Na^+^-selective channelrhodopsins (NaChRs) provide specificity in ion conduction. By predominantly allowing Na^+^ ions to pass, they minimize the unwanted effects of other ions. This specificity reduces crosstalk and ensures that the observed cellular responses are primarily due to Na^+^ influx, making experimental results more reliable and easier to interpret [5].

Finally, the influx of Na^+^ ions through NaChRs generates strong depolarizing currents in excitable cells. This high conductance ensures that fewer channels need to be activated to achieve a significant electrical response. This efficiency is particularly useful in optogenetic applications where robust activation is required with minimal light exposure [6,7].

NaChRs can be used in combination with other optogenetic tools that control different ions or pathways. This combinatorial approach allows for more sophisticated manipulation and interrogation of cellular and neural functions, enabling researchers to study complex biological processes with greater precision [8,9].

As such, this paper will focus on listing the available sodium channelrhodopsins together with their usefulness for optogenetic manipulations.

## 2. A Brief Natural History of Channelrhodopsins in General

**Figure 1:**
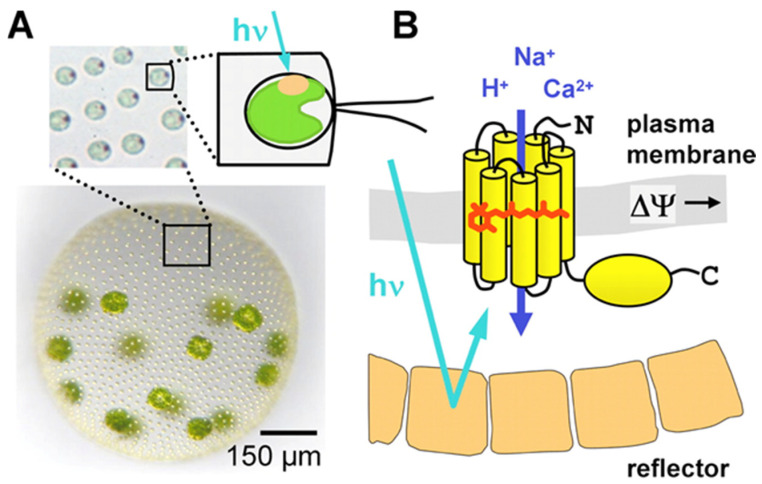
(**A**) Volvox colony with a few thousand small Chlamydomonas-like somatic cells and 16 reproductive gonidia. The inset shows a few enlarged somatic cells with their red eyes. The cells at the surface of the colony with their flagella are shown schematically. (**B**) Volvox ChR in the eye spot is a light-gated cation channel, which is composed of a seven-transmembrane helix domain with the all-trans-retinal chromophore and a C-terminal tail of unknown function. Carotenoid-rich vesicles reflect the light and create a front-to-back contrast at the location of the ChR-photoreceptor. Absorption of light by Volvox ChR induces the opening of the channel and, thus, changes the membrane potential. This figure is reproduced with permission from Ernst et al. 2008 [13].

The ChRs (ChR1 and ChR2) were first discovered in the early 2000s in *Chlamydomonas reinhardtii*, a eukaryotic alga, where they function as phototaxis receptors by responding to light stimuli (Figure 1A). Their purpose is to allow ions to flow through the cell membrane and, consequently, for the algae to move [10].

Being the first known ChRs, ChR1 and ChR2, nonselective cationic ion channels discovered by Peter Hegemann and Georg Nagel, were intensely studied and characterized [11,12].

Structurally, ChRs are seven-helical transmembrane proteins that bind a light-sensitive molecule called retinal (Figure 1B). Upon absorption of photons, the retinal undergoes a conformational change, triggering the opening of the ion channel; this allows ions, predominantly protons (H^+^), sodium (Na^+^), and potassium (K^+^) ions, to flow across the membrane, generating an electrical signal (Figure 1B) [1,11].

**Figure 2:**
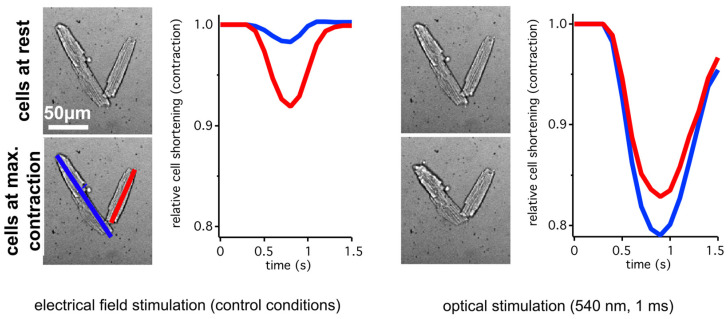
Application example of Channelrhodopsin 2 (ChR2). Isolated cardiac myocytes from a mouse with cardiac expression of ChR2 show contraction after being electrically paced (**left**) and light stimulated (**right**). The colored graphs present the cell length of two individual cells as indicated in the leftmost lower image. As seen in the extent of the induced cell shortening, optical stimulation was more effective compared to electrical field stimulation. The figure has been adapted with permission from Kaestner et al. 2018 [15].

The discovery of these specific ChRs led to an insurgence in the field of optogenetics, mostly used for neuroscientific applications, as optogenetics encompasses the integration of genetic and optical control to achieve gain or loss of function of precisely defined events within specified cells of living tissue [14]. An example of ChR application in isolated cardiac myocytes is outlined in Figure 2 which shows cardiac excitation-contraction after induction of an action potential through the use of light-activated ChR2.

**Figure 3:**
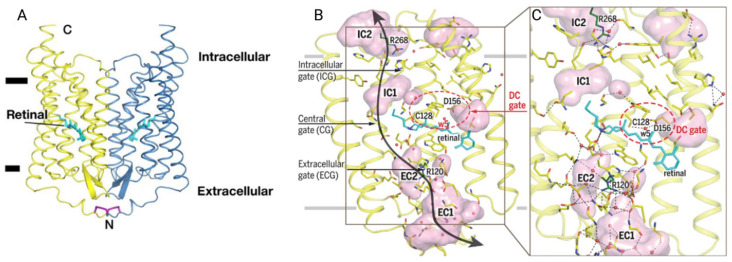
(**A**) Overall structure presentation of the Channelrhodopsin 2 (ChR2) dimer. Cysteine bridges are shown in purple. The retinal is shown in light blue. (**B**,**C**) General structure presentation of the ChR2 protomer. (**B**) Four cavities and three gates form the channel pore. (**C**) Extended hydrogen-bond network. The “DC gate” is shown in the red dashed ellipse. The black arrows and gray horizontal lines show the putative ion pathway and position of hydrophobic/hydrophilic boundaries, respectively. The figure has been adapted with permission from Volkov et al. 2017 [19].

First thought to be an H^+^ conductive channel, ChR1 was expressed in *Xenopus laevis* oocytes and discovered to be permeable to other cations such as Na^+^, K^+^, and Ca^2+^ [16]. This multi-ion permeability, though, is much lower than ChR2: in fact, ChR2 has a higher conductance to cations such as Na^+^, K^+^, and Ca^2+,^ while ChR1 is mostly H^+^ permeable. This difference, together with diverse light absorption properties, causes ChR1 to be unsuitable for controlling neuronal excitability because the number of protons that permeate the channel is insufficient to depolarize neurons above the threshold at physiological pH [17]. As such, ChR2’s biophysical properties have been studied more intensively and genetic modifications and protein engineering have expanded the spectral and kinetic characteristics of the ion channel, enabling more precise control over cellular activity and overcoming the limitations of this already useful optogenetic instrument [18]. The main structure of ChR2 can be found in Figure 3A. ChR2 is represented as a dimer with the retinal highlighted in blue. Figure 3B shows the ChR2 monomer and its gating system, allowing a clear view of the path the ions should take once the retinal undergoes its physical conformational change due to light irradiation.

## 3. Properties of Channelrhodopsins

Channel conductance, one of the main biophysical properties of ChRs, is of paramount importance as it directly influences the efficacy of light-induced depolarization in the cell membrane. A high conductance correlates with a substantial photocurrent, indicating that the greater the channel’s conductance, the fewer channels need to open to achieve a significant electrical effect. For example, Phototaxis Channel Rhodopsin (PsChR), a receptor found in the alga *Platymonas* (*tetraselmis*) *subcordiformis*, exhibits a unitary conductance that is three times higher than typical ChR2, resulting in a correspondingly higher current amplitude [7,18].

The kinetics of the opening and closing of ChRs are critical determinants for achieving precise temporal control of membrane potentials. The rate of membrane potential modulation induced by ChRs is directly linked to their channel kinetics. This is particularly important when utilizing ChRs to stimulate neural activity, as elucidating brain function necessitates technologies capable of manipulating the activity of large neuronal populations with cellular resolution and sub-millisecond precision. Although the ideal objective is to attain the fastest possible kinetics, this requirement must be balanced with light sensitivity. Enhancements in light sensitivity are often accompanied by slower kinetics. The ChR variants such as Chronos, CheRiff [20], and *Ps*CatCh2.0 [21,22], along with their mutations and derivatives, successfully strike a balance between these opposing factors, advancing research towards more precise and efficient neural control [18,23].

Spectral response is another key factor, as the wavelength dictates the extent of light penetration into the target site. Most ChRs exhibit a blue-lighted absorption spectrum, such as ChR2 whose peak excitation is at 470 nm [18]. However, mutations that alter the spectral properties towards a bathochromic shift to enable deeper tissue penetration have been developed, expanding the potential applications in the field of optogenetics. For example, Volvox ChR1 is a red-shifted ChR variant derived from *Volvox carteri*. Its longer wavelength activation, with peak absorption at 530 nm, allows deeper tissue penetration and reduces light scattering, which is advantageous for in vivo experiments involving deeper brain regions [24]. Chrimson, a ChR from *Chlamydomonas noctigama* is the variant with the most red-shifted absorption peak at 590 nm, which is more than 40 nm further red-shifted than all other ChRs. This permits even deeper light penetration [18,25,26].

Finally, ion selectivity is an ever-evolving focus of ChR variants, as it determines the type and extent of cellular responses ChRs can mediate [18]. When activated by light, conventional ChRs depolarize the membrane by conducting protons as well as monovalent (Na^+^, K^+^) and divalent cations (Ca^2+^, Mg^2+^) [11,12]. Since classical ChRs like ChR2 cause depolarization through cation conductance, over time, anion ChRs (ACRs) have been naturally discovered or genetically developed, allowing for membrane hyperpolarization through light-gated anionic conduction [27,28,29].

Chloride (Cl^−^) ChRs are a specialized class of anionic ion channels designed to selectively conduct Cl^−^ in response to light stimuli. These channels are significant for optogenetic applications that require neuronal inhibition, as the influx of Cl^−^ hyperpolarizes the cell membrane, reducing neuronal excitability [27,30,31].

Furthermore, as a long-awaited inhibitory tool, ChRs with highly-specific potassium permeability, such as *H. catenoides* (HcKCR1) and WiChR [32], have been discovered, which allows for powerful hyperpolarization to suppress excitable cell firing upon illumination, and to study and potentially treat K^+^ channelopathies [33].

Calcium (Ca^2+^) permeable ChRs [5,34,35] with reduced Na^+^ and H^+^ conductance and strongly improved Ca^2+^ permeation are highly advantageous for studying the role of Ca^2+^ as a second messenger. These channels have been engineered or discovered to facilitate precise control over Ca^2+^ signaling in cells, which is critical for various Ca^2+^-dependent physiological processes such as neurotransmitter release, gene expression, and synaptic plasticity in neurons [34] or excitation-contraction coupling in cardiac myocytes [36].

Lastly, NaChRs are used to generate strong depolarizing currents in excitable cells, such as to activate neural pathways [2,7,37].

## 4. Sodium Permeable Channelrhodopsins

NaChRs represent pivotal tools in optogenetics, primarily employed in neuroscientific research but with potential applications extending into other scientific fields. Since naturally occurring Na^+^-selective ion channels have not been identified (for example, HcChR displays higher Na^+^ permeability but lacks exclusivity to Na^+^ ions), researchers have addressed this gap by engineering mutations or creating variants of existing ChRs. These modifications aim to enhance Na^+^ conductance and selectivity over other ions [2,3,38].

Most NaChR variants focus on facilitating the influx of Na^+^ ions into the cell. However, some light-driven Na^+^ pumps (NaRs) are designed to extrude Na^+^ from the cell [39,40]. Although NaRs are not technically classified as ChRs due to their ion-pumping mechanism that utilizes light energy, they can be genetically modified to function ChRs, opening up new pathways for optogenetics [41].

### 4.1. PsChR

The first discovered ChR with an increased Na^+^ conductance was PsChR. PsChR is obtained from the phototactic alga *Platymonas* (*tetraselmis*) *subcordiformis* [42]. First discovered in 2013, its primary structure contains 660 amino acids, and its N-terminal half forms the 7TM (rhodopsin) domain. The domain demonstrates light-gated channel activity typical of other high-efficiency ChRs. The action spectra of PsChR-generated currents are measured using a 50 ms light pulse of low intensity. The maximum spectral activity is found at 445 nm, which makes PsChR the shortest wavelength-absorbing ChR characterized at the time. PsChR has been expressed in HEK293 cells by transfecting the cells with the mammalian expression vector that contains PsChR cDNA encoding the 7TM domain (amino acids residues 1–326) [7,42].

The mean peak current generated by the ChR at saturating light intensity is 4.6 ± 0.4 nA, compared with 2.5 ± 0.2 nA for ChR2. The difference between the plateau currents for these two ChRs is even greater: ~three-fold [7].

Current inactivation, calculated as the difference between the peak and plateau current relative to the peak current, is ~1.5-fold less for PsChR than for ChR2. Peak recovery is also studied by delivering a second light pulse after a short dark interval following the first pulse. The recovery of the peak current generated by PsChR is faster than that of any ChR tested so far. In particular, 50% of the initial peak amplitude is recovered in a ~30-fold shorter time than with ChR2 measured under the same conditions. Finally, ion selectivity reveals that inward photocurrents generated by PsChR are almost entirely carried by Na^+^ ions. This is proven by the experiment that the partial replacement of Na^+^ in the bath with nonpermeable organic N-methyl-d-glucamine led to the suppression of the inward currents. Later, PsChR was expressed in cultured hippocampal neurons to test whether it is relevant for optogenetic applications. Neurons expressing PsChR are capable of firing action potentials upon light stimulation with brief light pulses at frequencies up to 50 Hz, which is the upper limit for pyramidal neurons [43]. Thus, PsChR can be used for optogenetic applications. Together with its faster recovery time and its blue-shifted spectrum, PsChR is ideally suited for coupling with long wavelength absorbing rhodopsin pumps for light-regulated bidirectional control of the membrane potential or with fluorescent voltage- or Ca^2+^-sensitive probes, which are becoming increasingly popular [4,7,36].

### 4.2. PsChR Variants

While PsChR is already an effective ChR, its properties have been enhanced through targeted mutations. One notable variant is PsChR D139H, created by substituting the aspartic acid of the transmembrane helix 4 at position 139 with histidine. This mutation improves photocurrent amplitude, increases Na^+^ conductance, and reduces the permeability to other ions. The D139H mutation enhanced the P_Na+_/P_H+_ to 3.5, in comparison to the 1.2 of PsChR (Table 1). It exhibits a substantial reversal shift (mean ± SEM: 91 ± 2.2 mV) upon Na^+^ concentration change and also an enhanced permeability ratio of Na^+^ to H^+^ (P_Na+_/P_H+_ = 90 × 10^−7^) [6,7,44,45,46]. Further enhancement of PsChR D139H can be achieved through LR fusion, which involves adding a 17-amino-acid leucine-rich (LR) signal peptide to the cell culture containing the ChR. All LR peptides are fused to the N-terminal of the ChR, resulting in a three to four-fold increase in photocurrent and significantly higher expression levels in the cell membrane compared to non-fused ChRs. Another method to augment photocurrent amplitude is the truncation of amino acids at the N-terminal adjacent to the leucine-rich peptide sequence. Various truncations have been tested, and while some yielded negative results, a truncation of 19 amino acids at the N-terminal demonstrates increased photocurrent. This modified ChR, featuring both N-terminal truncation and LR fusion, is termed PsChR D139H 2.0 [6,7,44,45,46].

As such, these mutations allow for an improvement of optical tools based on PsChR, opening the path to further variants and better characteristics.

### 4.3. HcCCR

HcCCR is another newly discovered ChR. First located in 2018 in the genome of *Hyphochytrium catenoides* [47], and then reconfirmed in 2022 [48], this NaChR forms, together with two K^+^ selective ChRs (HcKCRs), a unique highly homologous group of light-gated ion channels with K^+^/Na^+^ permeability ratios differing over a wide range [33].

HcCCR is first characterized by expressing it in HEK293 cells. The reverse potential (V_rev_) estimated by approximation of the I-V-curve to zero current, is >40 mV. The P_K+_/P_Na+_ value, calculated from the V_rev_ shift using the Goldman–Hodgkin–Katz equation, is ~0.2. This value is extremely small compared to previous ChRs, thus, proving the Na^+^ selectivity of HcCCR. The maximal spectral sensitivity is found at 530 nm [2].

HcCCR is further characterized by a smaller relative permeability to H^+^ compared to Na^+^ than that of typical chlorophyte cation ChRs, and so is potentially useful as an optogenetic tool for neuronal activation that is less likely to produce undesirable acidification of the cytoplasm. HcCCR comes in conjunction with two K^+^ ChRs, HcKCR1 and HcKCR2, that were discovered in the same genome [2,49].

The existence of such closely related proteins such as HcKCRs and HcCCR, with such dramatic differences in the K^+^ to Na^+^ permeability ratio, provides a unique opportunity for structure-function analysis and allows for an in-depth comparison to further understand what makes a ChR permeable to a specific ion. While the researchers in the article [2] focus on analyzing the mechanisms that lead to K permeability—the results show that residues responsible for the K^+^ selectivity of HcKCRs are located in TM2 and TM7—a study could be made to figure out what makes HcCCR Na^+^ permeable compared to HcKCRs and possibly replicate in other ChRs, thus, further improving Na^+^ selectivity and allowing an increase in the usefulness of these optogenetic tools.

## 5. NCR1

An example of NaChR application is shown in [8] which primarily focuses on the Na^+^-selective ChR NCR1 2.0. Notably, NCR1 2.0 and HcCCR are originally from the same ChR but different in amino acid codon bias, sequence length and terminal modifications. With the two ChRs sharing the majority of characteristics and properties, the main difference can be found in the amino acid sequence. HcCCR encompasses the 1–265 amino acid region of the full genomic sequence, while HcNCR1 utilizes the 11–283 region and includes several plasma membrane targeting-enhancing peptides [8,48]. Furthermore, NCR1 2.0, as PsChR D139H 2.0, has the LucyRho signal peptide (LR) fused to the N-terminus to enhance its expression in the plasma membrane. This modification, alongside a truncation in the primary sequence, results in an 11-fold increase in photocurrent, making NCR1 a more effective optogenetic tool through further genetic manipulation.

The article [8] explores an innovative optogenetic approach to regulate water transport across cell membranes using the combination of light-gated anion channels, aquaporins (AQP), and light-gated Na^+^ or K^+^ channels.

This combination allows precise control over osmotic gradients, which, in turn, regulate water flux across membranes. By selectively activating different ion channels with light, it is possible to control the direction and rate of water flow. This bi-directional control is demonstrated by expressing mammalian AQP1 in *Xenopus* oocytes and observing morphological changes under different osmotic conditions [8]. This research represents a significant advancement in optogenetics, showcasing the potential of ion-selective ChRs to regulate vital physiological processes through controlled water transport.

## 6. Light-Driven Sodium Pumps (NaRs)

As mentioned before, in addition to the NaChRs, Na^+^-pumping rhodopsins (NaRs) are a class of microbial rhodopsins that actively transport Na⁺ across cellular membranes in response to light [40]. Upon absorbing light, NaRs undergo conformational changes that drive the translocation of Na⁺ ions from the cytoplasm to the extracellular environment, contrasting with the inward Na⁺ flux mediated by NaChRs. This outward Na⁺ transport contributes to maintaining ion gradients that are essential for various cellular processes. A notable example is the NaR from *Krokinobacter eikastus* (KR2) [39], which has been extensively studied for its structure and ion-transport mechanisms. Research into NaRs not only enhances our understanding of microbial rhodopsins but also holds the potential for developing optogenetic tools for precise control of cellular Na⁺ dynamics.

NaRs, as ion pumps, are significantly less efficient than ion channels and are, therefore, not ideal as optogenetic tools for robust control. However, the discovery of additional NaR subgroups provides a foundation for rational optimization of Na⁺-manipulating optogenetic tools [49], offering the potential for bi-directional control of Na⁺ flux.

## 7. A Putative Outlook

HcCCR and PsChR, thus, represent the two main variants of NaChRs that have been discovered or engineered up until now and while applications are still sparse and mainly limited to neuroscience applications, we envision novel applications in cancer research and in the context of red blood cells that could contribute to the development of a new drug delivery system. Both concepts are detailed below.

### 7.1. Cancer Research

NaChRs might be used as instruments to investigate and hence better understand cancer cell migration and invasion. Na^+^ plays an important role in the formation of invadopodia, specialized adhesive protrusions that motile cancer cells to migrate and cross into new environments, a process that is essential for metastasis [50]. Invadopodia has been investigated in various cancer cell lines, derived from different tissues such as breast, prostate, and head [51]. They function by releasing lytic enzymes and while this is not sufficient to degrade the extracellular matrix, the acidification of the surrounding environment, coupled with the reduction of bonds in the extracellular matrix, allows for the proliferation of cancer cells in new environments. Their presence is directly connected to metastasis formation [52,53].

Recent studies have elucidated a connection between Na^+^ dynamics and invadopodia formation. Voltage-gated Na^+^ channels have been found to be strongly upregulated and conserved across most cancer cells, suggesting a role in the propagation of the disease. An increase in Na^+^ channel expression results in increased cancer cell motility, invasiveness, and metastatic potential compared to cancer cells that do not express these ion channels. Their presence in cells containing invadopodia allows for improved proliferation due to the role they are thought to play in assisting the degradation of the extracellular matrix [53,54].

As such, investigating and manipulating Na^+^ signaling using NaChRs, or ideally a combination of NaChRs with efficient NaRs, in these cells could be a useful tool to learn about cancer invasion in general and the action of invadopodia in particular. While some aspects have been answered, there are still a lot of open questions as to how Na^+^ affects cancer cells and NaChRs could indeed be an instrument used to finely control Na^+^ influx and, thus, enable research to better understand the interaction between Na^+^ and cancer cells.

### 7.2. A Red Blood Cell-Based Drug Delivery System

Red blood cells are different from other eukaryotic cells [55]. Unlike most other eukaryotic cells, red blood cells maintain a membrane potential around −12 mV, caused by the low K^+^ (and Na^+^) permeabilities and determined mainly by Cl^−^ ions [56,57].

While an increase of the K^+^-permeability in red blood cells, e.g., by activating the Gárdos channel (KCNN4), leads to cell shrinkage [58], an increase of the Na^+^ permeability leads to red blood cell swelling, as can be seen, e.g., for the activation of the non-selective ion channel TRPV2 [59,60]. In the case of TRPV2 activation, the Na^+^ uptake and the associated swelling over-compensates for the shrinkage induced by K^+^ loss.

If a NaChR were to be expressed in red blood cells, and subsequently be activated through external light, the Na^+^ flux is expected to lead to a red blood cell swelling that is big enough to let the cells burst and release any content they may be carrying.

Due to the fact that red blood cells lack a nucleus and, as such, translational machinery, NaChR needs to be expressed on the level of precursor red blood cells [61]. We propose to perform such an expression in vitro and then upscale the production of the genetically modified red blood cells in bioreactors [62], a process that in principle has already entered clinical trials [63] and can be used for future applications.

Moreover, another key component of this scenario is the therapeutic drug that will be released inside the human body. To load the drug inside the red blood cell, we would like to refer to the red blood cell property that allows them to become so-called ghosts. Ghosts are essentially burst red blood cells that have lost their haemoglobin but are resealed to still form a membrane-coated container [64,65]. As such, the drug can be loaded in place of the haemoglobin, thus, making the ghosts effective drug carriers. This property has been explored and used in the past to load red blood cells with cargo such as potential drugs [66,67] which could be applied to the in vitro-generated red blood cells. Once a therapeutic drug has been loaded, the NaChR-containing red blood cells will function as a drug delivery system [68].

To this purpose, the goal of this use case is to load a drug into a NaChR-containing red blood cell, inject it into the human body and, once in loco, activate the ChR through external light allowing for a localized drug release. An advantage of this system is that the irradiation of light can be targeted to a specific zone, circumventing peripheral damage that the drug may cause if it is injected directly into the circulatory system [68,69].

In addition, there are several advantages to using red blood cells as carriers in the drug delivery system, but the principal one is that they are a native part of the human body and, as such, will not be rejected once injected into the bloodstream [68]. This will allow for a functioning drug-delivery system that might be used for different medical applications. 

## Figures and Tables

**Table 1:** Properties of channelrhodopsins and variants. (n.a. stands for not available) * NCR1 2.0 and HcCCR are originally from the same ChR but different in amino acid codon bias, sequence length and terminal modifications.

ChR Variant	λ_max_ (nm)	Current Inactivation	Current Peak Recovery	Reversal Potential Shift (mV)	Permeability Ratio (Na^+^/K^+^)	Permeability Ratio (Na^+^/H^+^)
ChR2	470	32%	50% after 5 s	24 ± 0.6 for Na^+^ 20 ± 0.7 for H^+^	1.2	3.1 × 10^−7^
PsChR	445	50%	50% after 0.15 s	58 ± 2.8 for Na^+^ 8.8 ± 5.5 for H^+^	1.2	18 × 10^−7^
PsChR D139H	445	n.a.	50% after 0.15 s	91 ± 2.2 for Na^+^ 5.5 ± 0.6 for H^+^	3.5	90 × 10^−7^
HcCCR	530	0–21%	n.a.	n.a.	5	n.a.
NCR1 2.0 *	516–540	0%	n.a.	n.a.	5.8	n.a.
